# Autophagy and Mitophagy in Diabetic Kidney Disease—A Literature Review

**DOI:** 10.3390/ijms26020806

**Published:** 2025-01-18

**Authors:** Alina Mihaela Stanigut, Liliana Tuta, Camelia Pana, Luana Alexandrescu, Adrian Suceveanu, Nicoleta-Mirela Blebea, Ileana Adela Vacaroiu

**Affiliations:** 1 Clinical Medical Disciplines Department, Faculty of Medicine, Ovidius University of Constanta, 900470 Constanta, Romania; alina.stanigut@365.univ-ovidius.ro (A.M.S.); tuta.liliana@univ-ovidius.ro (L.T.); alexandrescu_l@yahoo.com (L.A.); adrian.suceveanu@univ-ovidius.ro (A.S.); 2 Nephrology Department, County Emergency Clinical Hospital of Constanta, 145 Tomis Street, 900591 Constanta, Romania; 3 Gastroenterology Department, County Emergency Clinical Hospital of Constanta, 145 Tomis Street, 900591 Constanta, Romania; 4 Department of Pharmacotherapy, Faculty of Pharmacy, Ovidius University of Constanta, Aleea Universitatii Nr. 1, 900470 Constanta, Romania; 5 Department of Nephrology, Carol Davila University of Medicine and Pharmacy, 020021 Bucharest, Romania; ileana.vacaroiu@umfcd.ro; 6 Department of Nephrology, Sf. Ioan Clinical Emergency Hospital, 042122 Bucharest, Romania

**Keywords:** autophagy, mitophagy, diabetic nephropathy, PINK1/Parkin pathway, AMPK-mTOR-Sirt1 pathway, mitochondrial dysfunction

## Abstract

Autophagy and mitophagy are critical cellular processes that maintain homeostasis by removing damaged organelles and promoting cellular survival under stress conditions. In the context of diabetic kidney disease, these mechanisms play essential roles in mitigating cellular damage. This review provides an in-depth analysis of the recent literature on the relationship between autophagy, mitophagy, and diabetic kidney disease, highlighting the current state of knowledge, existing research gaps, and potential areas for future investigations. Diabetic nephropathy (DN) is traditionally defined as a specific form of kidney disease caused by long-standing diabetes, characterized by the classic histological lesions in the kidney, including mesangial expansion, glomerular basement membrane thickening, nodular glomerulosclerosis (Kimmelstiel–Wilson nodules), and podocyte injury. Clinical markers for DN are albuminuria and the gradual decline in glomerular filtration rate (GFR). Diabetic kidney disease (DKD) is a broader and more inclusive term, for all forms of chronic kidney disease (CKD) in individuals with diabetes, regardless of the underlying pathology. This includes patients who may have diabetes-associated kidney damage without the typical histological findings of diabetic nephropathy. It also accounts for patients with other coexisting kidney diseases (e.g., hypertensive nephrosclerosis, ischemic nephropathy, tubulointerstitial nephropathies), even in the absence of albuminuria, such as a reduction in GFR.

## 1. Introduction

Autophagy and mitophagy are critical cellular processes that maintain homeostasis by removing damaged organelles and promoting cellular survival under stress conditions. In the context of diabetic kidney disease, these mechanisms play essential roles in mitigating cellular damage.

**Figure 1:**
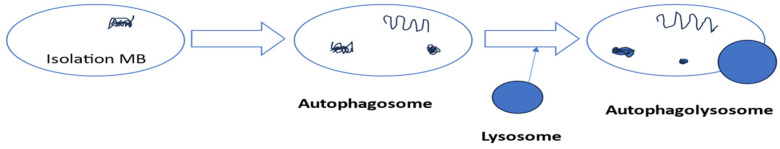
Molecular mechanism of autophagy: cytosolic components are included in autophagosomes. The autophagosome fuses with the lysosome becomes autophagolysosome and then degrade the cytosolic components.

**Autophagy** is a highly conserved cellular process where cells degrade and recycle their own components. It plays a vital role in maintaining cellular homeostasis (lysosome-dependent), especially in stress conditions by eliminating damaged organelles and proteins, glycogen, and accumulated lipid particles. There are three types of autophagy: macroautophagy, microautophagy, and autophagy mediated by chaperone (CMA). In the case of macroautophagy or simple autophagy, cytosolic components are included in a vesicle with a double membrane—autophagosome. The autophagosome fuses with the lysosome becomes an autophagolysosome and then degrades the cytosolic components (Figure 1). In microautophagy, the cytosolic components are included directly in lysosomes by a process of membrane invagination [1,2]. In the last type of autophagy, the lysosome is identified by the membrane protein (LAMP)-2A and eliminates heat shock protein of 70 or 90 kDa (hsc70 or hsc90) which are recognized by proteins with KFERQ motifs [2,3]. Authophagy-related genes (Atg) ensure the molecular regulation of autophagy. Deletion of Atg genes in nephrons, tubules, podocytes, and endothelial cells induces an abnormal functional response and structural abnormalities in the kidney. The process of autophagy is stimulated by different factors: hypoxia, oxidative stress, starvation, and cytokines: tumor necrosis factor (TNF)-α, interferon (IFN)-α, and DAMPs damage-associated molecular patterns [4,5].

**Mitophagy** is a specific form of autophagy that focuses on the removal of damaged mitochondria. In kidney diseases, mitophagy is crucial for maintaining mitochondrial quality control, which is important due to the high energy demands of renal cells. There are 3 types of mitophagy: type 1—triggered by nutrient limitation, type 2—determined by damage signals, and type 3—micro-mitophagy–related to small mitochondria-derived vesicles. Type 1 and 2 of mitophagy implied the inclusion of mitochondria into an autophagosome [6]. The studies of Um et al. and Sun et al. [6,7,8] made in vitro and in vivo in fluorescent transgenic mouse models demonstrate that mitophagy plays an important role in normal development. Two signaling pathways of mitophagy are described: the PINK1/Parkin pathway and mitophagy receptor pathway [9]. The mitophagy receptor has three components: outer mitochondrial membrane (OMM) proteins, mitochondrial membrane lipids—for example, cardiolipin [9,10] and mitophagy receptors—for example, a component of inner mitochondrial membrane (IMM) called prohibitin2 (PHB2) described by Wei et al. [11]. The outer mitochondrial membrane (OMM) proteins described are: FK506 binding protein 8 (FKBP8), FUN14 domain containing 1 (FUNDC1), autophagy/beclin 1 regulator 1 (AMBRA1), Bcl-2-like protein 13 (Bcl2-L-13), BCL-2/adenovirus E1B 19-kDa interacting protein 3 (BNIP3), Bcl-2/E1B-19K-interacting protein 3-like (BNIP3L/NIX) [9].

### 1.1. The PINK1/Parkin Pathway of Mitophagy

PINK1 is a serine/threonine kinase that has a mitochondrial target sequence (MTS) and a transmembrane domain (TMD). Parkin is a cytosol ubiquitin E3 ligase.

In normal mitochondria, PINK1 is transported into the IMM by the TOM (translocase of the outer membrane)/TIM (translocase of the inner membrane) complex. The transport is made by a mitochondrial membrane potential-dependent process [12,13,14]. A peptidase–mitochondrial processing peptidase (MPP) splits the MTS and TMD is split by resenilin-associated rhomboid-like protease (PARL) [13,14,15]. After these cleavages remains a 52kDa fragment which contains the kinase domain of PINK1. The fragment is exposed to the cytosol until its degradation [13,14].

**Figure 2:**
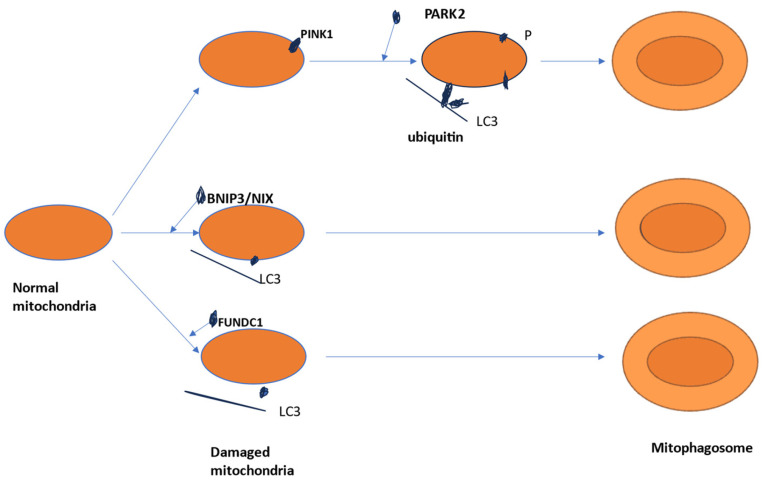
Molecular mechanisms of mitophagy: there are three well-described pathways of mitophagy: PINK1-PARK2 pathway, BNIP3/NIX receptor pathway, and FUNDC1 receptor pathway.

In affected mitochondria PINK1 is not transported anymore and, as a consequence, full-length PINK1 is accumulated on the OMM [13,16]. PINK1 kinase is activated by auto-phosphorylation [14,16,17]. Phosphorylated PINK1 [14,18] phosphorylates ubiquitin on serine 65 (Ser65), leading to the passage of Parkin from the cytoplasm to the damaged OMM and its partial activation [9,19,20]. Figure 2.

Parkin translocates phospho-Ser 65-ubiquitin (PSER65-UB) to OMM proteins or the ubiquitin substrate, which leads to more phosphorylated substrates for PINK1 and induces a positive feedback loop [9,19].

### 1.2. Mitophagy Receptor Pathway

Mitophagy can directly be induced by mitophagy receptors (containing LC3-interacting region-LIR motif) localized in the OMM and IMM (Figure 2). Different studies demonstrate that mitophagy is promoted by specific OMM mitophagy receptors such as BNIP3 and BCL2 interacting protein 3 like (BNIP3L/NIX [14,21], FUN14 domain-containing protein 1 (FUNDC1) [14,22] and FKBP prolyl isomerase 8 (FKBP8) [14,23]. In hypoxia, BNIP3 and NIX are activated by HIF—hypoxia-inducible factor-1 and bridge mitochondria with LC3 in the autophagosome membrane and form mitophagosome [24,25]. One study by Zhang [26] shows that NIX interacts with small GTPase and can activate mitophagy [25,26], linking mitochondria to autophagosome by interacting its LIRs domains with LC3 [24,25]. The mitophagy induced by FUNDC1 depends on the phosphorylation of this mitophagy receptor. In normal conditions, FUNDC1 (a new OMM protein involved in mitophagy under hypoxia) is phosphorylated by the protein kinase SRC and stops interaction with LC3. Hypoxia inhibits SRC activity and leads to a reduction in phosphorylation of FUNDC1 which has like consequences an increase in interaction between FUNDC1 and LC3 and promotes mitophagy [27,28,29].

A new type of mitochondrial receptor was described by Bhujabal et al. [23]—FKBP8 which belongs to a binding protein family—FK506. FKBP8 recruits LC3A to damaged mitochondria and promotes mitophagy in a different pathway like the PINK1-Parkin pathway.

To sum up, autophagy is a conserved intracellular process that recognizes and degrades damaged macromolecular proteins, organelles, and invading pathogens for cellular recycling (in order to maintain cellular homeostasis) while mitophagy is a selective form of autophagy that specifically removes damaged mitochondria [25]. Mitophagy is an adaptive or defense mechanism for maintaining a population of healthy mitochondria and ensuring cell survival [25].

### 1.3. Current State of the Art

#### 1.3.1. Key Theories on Autophagy and Mitophagy in Kidney Diseases

Before starting the exposure of mechanisms of autophagy and mitophagy in kidney diseases is important to present the most important instruments and methods for assessing autophagy and mitophagy in kidney diseases [7,8]:

#### 1.3.2. Instruments and Methods for Assessing Autophagy and Mitophagy in Kidney Diseases

1. Western blot for LC3 and p62: These proteins are commonly used markers for autophagy assessment. LC3-II is a key marker of autophagosome formation, while p62 indicates autophagic degradation [30].
2. Immunohistochemistry for PINK1 and Parkin: Used to assess mitophagy, particularly the PINK1/Parkin pathway that regulates mitochondrial quality control [31].
3. Electron Microscopy: Employed for visualizing autophagosomes and mitophagosomes in renal tissues, offering direct evidence of autophagy and mitophagy processes [9,26,28,29]. However, quantification frequently involves tedious and time-consuming morphometric analyses of a high number of cells in order to obtain statistically significant results.
4. Gene Expression Analysis for Autophagy-Related Genes: Quantitative PCR and mRNA expression analysis for autophagy genes like ATG5, ATG7, and BNIP3 are often used to assess autophagy activity in kidney cells [14].
5. Mitochondrial ROS Detection: Mitochondrial dysfunction is measured using ROS assays to detect oxidative stress in diabetic kidneys, often linked to mitophagy dysfunction [32].
6. Flow Cytometry for Apoptosis and Autophagy: Dual staining of cells for autophagic and apoptotic markers is used to assess crosstalk between cell death pathways in CKD [33].
7. Mitochondrial Membrane Potential Assays: JC-1 dye is commonly used to measure changes in mitochondrial membrane potential, a hallmark of mitophagy dysfunction [34].
8. MitoTracker Staining: This technique uses fluorescent dyes to label healthy versus damaged mitochondria, often used in mitophagy studies [6]. It is a flow cytometry-based approach to determine mitophagy by using MitoTracker Deep Red—MTDR (a mitochondria-selective probe). When used in combination with inhibitors of autophagy and mitophagy this method can determine mitophagy flux. This method has pitfalls due to the chemical properties of the MitoTracker dyes. MTDR staining can bind to and affect the activity of cysteine-containing proteins in mitochondria. This aspect can be partially avoided by performing the staining after treatments. Also, mitochondrial biogenesis can hide mitophagy assessment by MTDR, so this method should be used for short periods of time when there is mitophagy but not enough time for compensation by mitochondrial biogenesis. Another issue is that the probe reacts with thiols from selected proteins and changes that could affect the levels of those proteins or their redox status must be taken into consideration. Recently Jimenez-Loygorri et al. [35] described a standardized protocol to assess mitophagy in cells and ex vivo dissociated tissue using the tandem fluorescent mito-QC reporter via flow cytometry. The mito-QC reporter consists of a fusion protein containing mCherry-GFP-FIS1, that targets the OMM.
9. Autophagic Flux Assays: These assays, using inhibitors like bafilomycin A1, help assess the completion of the autophagy process by preventing lysosomal degradation [25].
10. High-Resolution Respirometry: Used to measure mitochondrial respiration and assess the functional consequences of mitophagy dysregulation in kidney diseases [36].

Autophagy can be quantified by a wide variety of methods: electron microscopy, Western blotting, and confocal fluorescence microscopy. Most of these methods are not applicable to high-throughput approaches. A recent method using a simple flow-cytometry-mediated quantification of the membrane (=autophagosome)-bound enhanced green fluorescence protein (eGFP)-LC3B is faster and allows the quantification of large numbers of cells at once. This method can be applied to answer different problems: pathway analysis, key factor identification, and drug discovery. There are also limitations of this method: autophagic flux versus accumulation of autophagosomes. For this reason, to support the conclusions of the initial screening methods, the screen should be complemented by monitoring the endogenous LC3B status in primary cells.

### 1.4. Autophagy and Chronic Kidney Disease

In CKD, autophagy is often impaired, contributing to cellular dysfunction and disease progression. The balance between autophagy activation and inhibition is critical, as excessive autophagy can cause cell death, while insufficient autophagy leads to the accumulation of damaged proteins and organelles. Lin et al. (2019) emphasize that autophagy dysfunction contributes to CKD by promoting oxidative stress and inflammation, and they suggest targeting autophagy as a therapeutic strategy [30]. Also, Han et al. (2023) discuss the role of autophagy in DN and CKD, focusing on its regulation through nutrient-sensing pathways and its potential for therapeutic interventions [37].

*Autophagic Flux in CKD*: In our days remains still unknown all the mechanisms of CKD. The final results of processes involved in CKD are glomerulosclerosis, tubulointerstitial fibrosis, and vascular sclerosis.

In CKD, autophagy plays a dual role, offering protection during acute kidney injuries but becoming maladaptive during chronic stages, and plays an important role in CKD. Patients with CKD have increased oxidative stress, elevated mitochondrial ROS production and, as a consequence, affected body homeostasis and inflammation [30,38,39,40]. Dysregulated autophagy contributes to inflammation and renal fibrosis [2]. It is very important to establish the autophagic status but, till now, there are no absolute criteria to be utilized in clinical or experimental studies to evaluate this status.

In 2013 Tien-An et al. [30,41] designed a method to evaluate the autophagic function in human leukocytes from patients with CKD. It was measured LC3-I (a cytosolic form of LC3 proteins)—considered to be an autophagosome marker and LC3-II (LC3-I conjugated to phosphatidylethanolamine)—reflected the autophagosome formation. It is known that LC3-1 is stable during starvation and the LC3-II level gives information about changes in the autophagic function and flow. So, LC3-I was considered to be an ideal control in human leukocytes and the ratio of the LC3-II versus the LC3-I (LC3-II/LC3-I) in leukocytes is an indicator of autophagy flux. As an indicator of autophagy flux or activation was utilized the ratio of LC3-II/LC3-I after 12 h of fasting versus LC3-II/LC3-I 2 h after breakfast and noted as γ LC3. The study enrolled 60 patients with CKD stages 4–5 (30 on hemodialysis and 30 without hemodialysis) and 30 healthy volunteers of similar sex and age in the study group. The autophagy flux in CKD patients was measured using γ LC3. The study results showed that autophagy flux and γ LC3 increased in overnight fasting only in healthy subjects and hemodialysis could not correct the autophagy flux deficiency in patients with CKD. After starvation, there was an increase in the Atg5, and Beclin-1 transcript levels only in the healthy group. The conclusions of this study were that there is strong evidence that CKD patients have impaired autophagy activation which was not reversed by hemodialysis [41].

The implications of autophagy in kidney fibrosis were studied in experimental rodent models—unilateral ureteral obstruction (UUO) or adenine diet. Treatment of cell culture with transforming growth factor (TGF)-β1 was used to evaluate the effects of increased fibrotic responses on autophagy. Initially, on days 3 and 7 after UUO, there was an increase in expression of LC3-II in the obstructed kidneys and a decline to almost basal level on day 14 [2,42,43,44]. The studies revealed that autophagy can be capable of both preventing and promoting kidney fibrosis [2,45]. Genetical inhibition of autophagy (by Knockdown of Becn1 or knocking out Map1lc3b or Becn1 or pharmacologically using 3-methyladenine—3MA) aggravated UUO or TGF-β1-induced fibrotic responses. Autophagy induced by knocking of Becn1 or rapamycin [2,43,46] decreased deposition of extracellular matrix (EMC) and expression of UUO-induced collagen type I. In mouse mesangial cells the production of TGF-β1-induced collagen type I is reduced by trifluoperazine-dependent stimulation of autophagy [2,47]. The study by Kim SI et al. [47] revealed that autophagy has antifibrotic functions by degrading intracellular collagen type I. There are also contradictory findings that show that obstructed kidneys from proximal tubule-specific Atg7-deleted mice presented nephron loss, reduced tubular atrophy, tubulointerstitial fibrosis, and macrophage recruitment after UUO [2,45]. Considering the results of this study we can say that UUO-induced kidney fibrosis is stimulated by persistent activation of autophagy.

*Interplay Between Autophagy and Cell Death*: Autophagy interacts with other forms of programmed cell death, such as apoptosis and necroptosis. This crosstalk determines the fate of renal cells under stress, with dysregulated autophagy tipping the balance toward cell death in CKD [48]. IL-1β and IL-18 synthesized by macrophages are important proinflammatory cytokines that induce NLRP3 (NLR family pyrin domain containing 3) inflammasome formation [2]. Activation of NLRP3 inflammasome induces apoptosis, necroptosis, and pyroptosis (the inflammatory form of programmed cell death)—Huang et al. [49]. Deficiency of LC3 and Beclin1 (autophagy regulators) increases synthesis (mediated by Caspase 1) of inflammatory cytokines with an accumulation of affected mitochondria in the macrophages. Caspase 1 stimulates pyroptosis. Excessive autophagy can cause apoptotic or necroptotic—dependent cell death [50,51]. Cell survival has the highest chance with autophagy followed by apoptosis and necroptosis [52]. The proteins from the BCL2 family regulate apoptosis (by increasing mitochondrial outer membrane permeabilization—MOMP) and suppress autophagy and PINK1/Parkin-dependent mitophagy [53,54]. The study by Lim et al. [55] showed that an affected autophagic process favored death in macrophages via receptor-interacting protein kinase (RIPK)1-dependent pathway and -independent pathway. The same study demonstrated that a defective autophagic response stimulates necroptosis by hyperactivated TLR and TNF signaling, so, autophagy prevents necroptosis. Another study by Nakahira et al. [56] demonstrated that autophagy prevents NLRP3-induced inflammation by maintaining mitochondrial integrity.

Choi et al. discuss how autophagy modulates inflammation and cell death, particularly in acute kidney injury and CKD, and highlight potential therapeutic strategies targeting autophagic pathways [48].

### 1.5. Role of Autophagy in Diabetic Kidney Disease

Autophagy is a key regulator of cellular homeostasis, particularly in kidney cells. The degradation of damaged proteins and organelles by autophagy ensures that cellular function is maintained during stressful conditions such as hyperglycemia. Studies have demonstrated that autophagy plays a protective role in various kidney diseases, including diabetic nephropathy. Impaired autophagy has been linked to the progression of DN and CKD, leading to increased cellular damage and fibrosis.

It is already known that renal cell injury in DN is induced (except oxidative stress) by 4 pathogenic mechanisms: generation of Advanced Glycation End products and activation of their receptors (AGEs-RAGEs), activation of polyol metabolic bypass, activation of protein kinase C (PKC), and overactivation of hexosamine bypass [33,57]. These 4 mechanisms interact with one another and, in addition to the intermediate products of the glycolytic pathway and superoxide products (the last one induced by accumulation of a high amount of reduced NADH/FADH2 product via the tricarboxylic acid cycle), accelerate the progression of kidney lesions [58,59]. There are described two ways leading to renal injury induced by oxidative stress and the four pathogenic mechanisms. In one way many balance systems (mitochondrial quality control, oxidation-antioxidant balance, protein homeostasis) are affected and in a second way, there is a regulation of the gene transcription and translation processes of intracellular DKD-related pathogenic Molecules (PKC activation and hexosamine pathway hyperactivation) [33].

**Figure 3:**
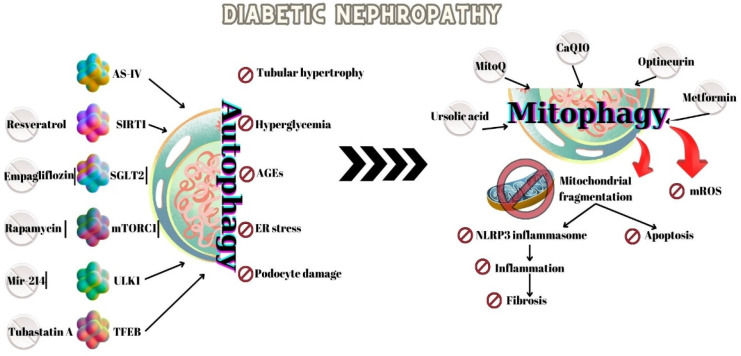
Autophagy and mitophagy in Diabetic Nephropathy and potential therapeutic targets. Autophagy prevents tubular hypertrophy, endoplasmic reticulum (ER) stress, accumulation of AGEs (advanced glycation end products), and loss of podocytes. Mitophagy inhibits mitochondria specific oxidative stress (mROS—mitochondria-derived reactive oxygen species), attenuates NLRP3 inflammasome-mediated tubular injury (NLR family pyrin domain containing 3), inflammation and fibrosis, AS-IV, astragaloside IV; CoQ10, coenzyme Q10; MitoQ, mitoquinone; mTORC1, mechanistic target of rapamycin (mTOR) kinase complex 1; SGLT2, Naþ-glucose cotransporter-2; SIRT1, sirtuin; TFEB, transcription factor EB; ULK1, uncoordinated-51-like protein kinase 1.

Autophagic activity and mitochondrial function are regulated by mTOR, AMPK, and NAD-dependent deacetylase sirtuin-1 (SIRT1). These molecules are involved in many pathways of development of type 2 diabetes and DN [60,61,62]—Figure 3.

*AMPK-mTOR-Sirt1 Pathway*: Autophagy in DN is tightly regulated by metabolic pathways, such as AMPK-mTOR and Sirt1, which respond to nutrient-sensing signals. Dysregulation of these pathways exacerbates kidney damage due to excess oxidative stress [37].

*mTOR pathway*: mTOR is a serine/threonine protein kinase, consisting of mTORC1 and mTORC2 and belongs to the PI3K-related protein kinase family. Its activity depends on intracellular nutrient levels and redox status [63]. mTOR inhibits intracellular autophagic activity and regulates related gene expression or protein modification [64].

The study by Sakaguchi et al. [65] showed that hyperglycemia induces mTOR activation and there is a strong correlation with proliferation and apoptosis of tubular cells in diabetic nephropathy and by knocking down the mTOR gene in proximal tubular cells there was an improvement in tubular injury—Table 1. Boutouja et al. and Ma MKM [66,67] demonstrated that Rapamycin—an mTOR inhibitor, ameliorates insulin resistance, increases blood glucose uptake, and prolongs the survival of species. Rapamycin is responsible for the improvement of tubular epithelial transdifferentiation to mesenchymal cells, glomerular basement membrane thickness, and macrophage recruitment and all these actions reduce proteinuria and ameliorate renal autophagic activity [67].

There is also an in vivo study by Tremblay et al. [68] showing that a high-fat diet increases insulin resistance by inhibition of glucose uptake and glycogen synthesis via overactivation of mTORC1 and inhibition of phosphorylation at the serine site of insulin receptor substrate 1 (IRS-1 Ser312 and Ser636 sites).

In the pathophysiology of DN, long noncoding RNAs (LncRNAs) also play an important role [37,69]. LncRNAs inhibit the autophagy-related Akt/mTOR pathway and affect the pathological alteration in the diabetic kidney [37,70].

*AMPK pathway*: AMPK is a serine/threonine protein kinase and is composed of 3 subunits: a catalytic subunit α and 2 regulatory subunits β and γ. AMPK becomes active after phosphorylation of threonine 172 (Thr172) located on the subunit α. In experimental models of diabetes AMPK expression and phosphorylation levels of AMPK were reduced in glomerular epithelial cells [33,71]. AMP/ATP ratio—an energy sensor, regulates AMPK [37]. In special conditions, like hypoxia and starvation, AMP/ATP ratio rises and AMP binds to the subunit γ of AMPK which leads to Thr172 phosphorylation [37,72]. Hormones, drugs, and proinflammatory cytokines can activate AMPK via TGF-β-activated kinase and CAMKKβ and induce autophagy in order to keep cellular energy homeostasis under starvation [37,72].

Recent studies [73,74,75,76] showed that in DKD with proteinuria and renal pathological alteration AMPK and autophagy are deactivated. Autophagy can directly be initiated by AMPK via phosphorylation of ULK1 or indirectly by blocking mTORC1 to release ULK1 [77]—Table 1. ATP synthesis decreases and AMP increases when blood glucose utilization decreases. Increased levels of AMP activate AMPK. Activated AMPK phosphorylates tumor suppressor (TSC2) and has GTPase activity transforming GTP to GDP. GDP inhibits mTOR via weakening the binding of GTP-dependent Rheb to mTOR molecules [37].

Kim et al. [78] demonstrated in a study that AMPK phosphorylates beclin1 at Thr388 induces dissociation of the beclin1/B-cell lymphoma-2 (Bcl-2) and initiates autophagy.

A recent study by Shati et al. [75] showed that AMPK activates Sirt1 (NAD-dependent deacetylase) by 2 mechanisms: increasing intracellular NAD+ levels and the second one: by phosphorylating and redistributing glyceraldehyde 3-phosphate dehydrogenase into the nucleus to free Sirt1 (also demonstrated by Chang et al. study) [79]. By these mechanisms, AMPK promotes autophagy and ameliorates DN.

All these data reveal that autophagy regulated by AMPK is very important in the development of DN and can be a target of the treatment for preventing DN.

*Sirt1 pathway*: Sirtuin family proteins are β-NAD+ or NAD+-dependent enzymes. SIRT1, 6, and 7 are principally localized in the nucleus, SIRT3, 4, and 5 are usually localized in mitochondria, and SIRT2 is present predominantly in the cytosol. SIRTs can modulate different cellular functions: DNA repair, mitochondrial biogenesis, apoptosis, oxidative stress, inflammation, glucose, and lipid metabolism [80,81]. All types of SIRT (except SIRT5) are involved in renal damage induced by diabetes. SIRT1, SIRT3, SIRT 4, SIRT 6, and SIRT 7 have renoprotective action in DN by regulating different cellular processes: autophagy, apoptosis, and fibrosis [81]. SIRT2 has a promoting effect on DN by increasing apoptosis and decreasing cell autophagy [81]. There is a close relationship between SIRT1 expression and renal function, in DN SIRT1 expression is suppressed in serum and renal tissues [81,82,83].

SIRT1 affects molecules associated with important signaling pathways: AMPK, and TGF-β1. AMPK activates SIRT1 and this activation is critical for regulating autophagy, apoptosis, and oxidative stress this is a conclusion of an in vivo study by Song et al. [84]. This study revealed that activation of the AMPK/SIRT1 pathway by STAMP2 increases autophagy and improves renal injuries in DN rats [81,84]. The study by Watanabe et al. showed that there is an activation of the mTOR pathway and a decrease in AMPK activation by inhibiting SIRT1 [85]. The result of inhibition of SIRT1 in this study was the activation of autophagy-related proteins—for example, Atg5 by deacetylation which stimulates autophagic activity. SIRT1 also activates the PINK1/parkin pathway and induces the synthesis of lysosomes and autophagosomes enhancing intracellular autophagy levels [33,86].

SIRT1 enhances autophagy by reducing acetylation or phosphorylation of different target genes: p53, AMPK, forkhead box O1 (FoxO1), and peroxisome proliferator-activated receptor-gamma coactivator-1α (PGC-1α) [81,87,88,89,90]. The study by Dong et al. showed that deacetylation of p53 by SIRT1 activates AMPK-dependent autophagy and ameliorates DN [91].

By deacetylating the transcription factor FoxO3 SIRT1 up-regulates Bcl-2/adenovirus E1V19-kDa interacting protein 3 (BNIP3) and increases autophagy and, therefore, inhibits DN [81,92,93,94]—Table 1.

There are studies that showed that different miRNAs: miR-135a-5p, miR-138, miR-150-5p, miR-155-5p, and miR-217 by targeting the 3′ untranslated region of Sirt1 neutralize the protective effect of SIRT1 against DN [37,91,95,96,97,98,99].

Yasuda et al. [30,100] revealed that in DN impaired autophagy in the kidney is associated with podocyte loss and massive proteinuria. In animal studies with diabetic mice Liu et al. and Ma et al. discovered that deficiency of autophagy activation by knockout Atg5 is responsible for severe proteinuria and renal failure [101,102]. Another animal study [103] with diabetic mice showed that autophagy can be activated by decreased mTORC1 activation, and ameliorates podocyte loss, glomerulosclerosis, and proteinuria, slowing down the progression of DN. In another study, Lenoir et al. reported that autophagy is activated in podocytes exposed to a high glucose concentration medium and avoided podocyte apoptosis [104].

Some studies revealed the opposite effect of a high glucose medium on podocytes. For example, the study by Xin et al. demonstrated that human podocytes exposed to a high glucose medium presented an important reduction in LC3-II and Beclin-1 and decreased autophagy activation [30,105]. Another study reported that a high glucose milieu induced podocyte apoptosis via CASP3 activation [106].

The relationship between podocyte-specific proteins—like podocin protein and autophagic markers was described in a few studies: an animal model—in the 50-week-old diabetic nephropathy rat model with severe proteinuria were found alteration of foot processes, reduction in LC3-II, reduction in podocin positive areas and p62 accumulation. The examinations of kidney biopsy samples of patients with DN and heavy proteinuria reported that podocin is expressed with an irregular and granular pattern under immunofluorescent examination and there is an important accumulation of p62 proteins in the glomeruli [30]. These results suggest that impaired autophagy can cause podocyte lesions in DN with important proteinuria [107].

Autophagy functions differently in various renal cell types. The normal function of podocytes requires a high level of autophagy [108]. In the early stages of DKD, there is an inhibition of autophagy in podocytes which leads to massive proteinuria and progression of diabetic nephropathy. Experimental models like Streptozocin (STZ)-induced DKD demonstrated that autophagy induces the degradation of cholesterol and ameliorates podocyte injury due to lipotoxicity and loss of autophagy is associated with disruption of the glomerular filtration barrier and glomerulosclerosis [108,109]. Another experimental model with a High-fat diet (HFD)-Induced Diabetes in Podocyte-Specific Autophagy-Deficient Mice reveals that impaired autophagy promotes podocyte loss and proteinuria. Lysosomal dysfunction also plays a role in podocyte injury knowing that lysosomes are involved in the processing of endocytosed albumin in podocytes [108].

In DN mesangial cells (MCs) are also protected by autophagy induced by TGF-β1 via TAK1 and PI3K-AKT-dependent pathways [103]. However, the role of autophagy in MCs in DN remains unclear [108]. High glucose stimulation of MCs suppresses autophagy-related protein levels: PINK1 and Parkin, mTOR, p62, and Beclin1 [108]. AGE-induced senescence in mesangial cells is accelerated by inhibition of autophagy [108,110,111] and induces renal inflammation and fibrosis in DN. AGEs-induced mesangial cell damage can be repaired by activation of MC autophagy [108]. There are needed further studies to establish the potential therapeutic value of autophagy in MCs in DN.

Regarding proximal tubular cells, hyperglycemia inhibits autophagy activation and increases P62 proteins in type 1 and type 2 diabetes animal models [107,112]. All the studies presented reveal that in DN there is a decreased autophagy activity and an increased apoptosis [30]. Apoptosis (type I programmed cell death), autophagy (type II programmed cell death, meaning “self-eating”), and necroptosis are 3 forms of programmed cell death that have been implicated in the pathogenesis of diabetic nephropathy (the major mechanisms were discussed above). Hyperglycemia generates reactive oxygen species synthesis and induces podocyte apoptosis. Apoptosis of podocytes induces podocyte depletion and glomerular injury which lead to proteinuria in diabetic nephropathy. Podocyte apoptosis starts with the onset of diabetes indicating that Hyperglycemia underlying the stimulation of pro-apoptotic signaling pathway. Also, podocyte apoptosis is mediated by p53 in cells exposed to high glucose. In diabetic nephropathy, apoptosis also affects the epithelial cells of the proximal convoluted tubules leading to tubular atrophy and the apparition of tubular glomeruli.

The renal proximal tubular epithelial cell (PTEC) lesions are important for the onset and progression of DN [108]. Unlike in podocytes, the basal level of autophagy in renal PTECs is very low [108] but the autophagy-lysosome pathway is important in PTEC. Also, impaired autophagy stimulates apoptosis and senescence of tubular cells. An experimental model with STZ-induced DKD knockdown of SGLT2 (sodium-glucose cotransporter 2—an important regulator of renal tubular glucose reabsorption) aggravates the impaired autophagy. Elevated AGEs induced by long-term HG levels suffer a process of endocytosis in PTEC lysosomes and accumulate with the PTEC autophagy impairment [108]. Accumulation of AGEs affects the lysosomal function and autophagic flux and aggravates PTEC injury.

Glomerular endothelial cell (GEC) dysfunction is mediated by high glucose levels, ROS accumulation, and autophagy inhibition and also plays an important role in the onset and development of DKD [108,113]. An experimental model with STZ-induced DKD deletion of Atg5 (a critical gene involved in autophagy) of GECs leads to capillary rarefaction and endothelial-to-mesenchymal transition and accelerated DN [108,114]. Tian et al. demonstrated in a study that activating the AMPK pathway ameliorated the renal injury of DN by improving the autophagy of GECs [108,115] and suggested the major role of autophagy of GECs in improving DN.

**Mitophagy in CKD.** The progression of CKD is delayed by the cytoprotective effects of mitophagy [2]. In experimental mouse models with kidney fibrosis and in human kidney biopsies with tubular atrophy and interstitial fibrosis were found a decreased expression of mitophagy regulators such as PINK1, Parkin, and MFN2 [116,117]. Bathia et al. [116] reported in a study that in patients with CKD there is a reduced expression of mitophagy receptors in peripheral mononuclear cells (PBMCs) and the conclusion of the study was that mitophagy has a protective effect against kidney fibrosis. The same study showed that the loss of Pink1 or myeloid-specific Mfn2 (which affects mitophagy) increased mROS production in kidney macrophages [116].

The implications of mitophagy in kidney fibrosis were demonstrated in experimental rodent models—unilateral ureteral obstruction (UUO) or adenine diet [116,117]. The studies of Li et al. [118] and Jin et al. [47] reported that there is a reduction in the expression of MFN2 and an increased PINK1 and Parkin expression in the kidneys after UUO. Mitophagy plays a protective role against oxidative stress, mitochondrial damage, and TNF-β1 expression [118] and regulates erythropoietin production by attenuating inflammation [119]. The kidney macrophage mitophagy response is affected by myeloid-specific ablation of Mfn2 [120]. In kidney fibrosis induced by an adenine diet, the mitochondrial fission proteins: DRP1 and phosphorylated DRP1 (which are implicated in the mitophagy process) are upregulated and the fusion proteins—OPA1, MFN1, and MFN2 are downregulated [120]. Activation of mitophagy by UMI-77 reduces renal fibrosis by reduction in apoptosis, inflammation, and activation of NF-kB and TGF-β/Smad signaling pathways [121]. Fibrosis induced by TGF-β1 in HK-2 cells is reduced by stimulation of mitophagy via UMI-77 [121]. Liu et al. [122] reported that Quercetin alleviates kidney fibrosis by reducing renal tubular epithelial cell senescence through the SIRT1/PINK1/mitophagy axis.

### 1.6. Role of Mitophagy in Diabetic Kidney Disease

Mitophagy, a selective form of autophagy that removes damaged mitochondria, is particularly important in diabetic nephropathy. Mitochondrial dysfunction, driven by oxidative stress and hyperglycemia, is a hallmark of DN. Studies show that proper mitophagy activity is critical for preventing mitochondrial damage and subsequent cellular apoptosis in kidney cells. Zhang et al. [9] provide evidence that mitophagy is a key process in maintaining mitochondrial quality in diabetic kidneys, highlighting its potential as a therapeutic target [9]. Yang et al. [123] highlight how mitophagy helps remove damaged mitochondria in tubular cells, protecting them from oxidative stress and apoptosis [123].

**Figure 4:**
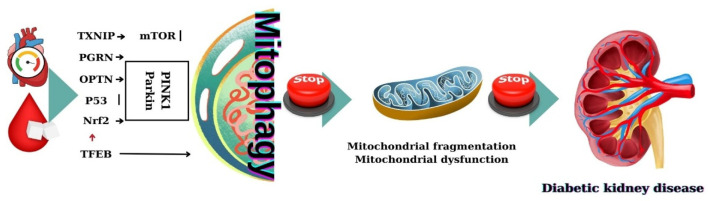
The potential mechanisms of mitophagy in diabetic nephropathy. Impaired mitophagy leads to an accumulation of damaged mitochondria which plays an important role in the pathogenesis of diabetic nephropathy.

Mitophagy dysfunction contributes to oxidative stress and inflammation in DN. Mitochondrial dysfunction is a critical factor leading to the development and progression of DN, and impaired mitophagy has been linked to the accumulation of damaged mitochondria in renal cells [9]—Figure 4.

The PINK1/Parkin pathway plays a central role in mitophagy regulation, especially under high glucose conditions seen in diabetes. This pathway helps prevent mitochondrial dysfunction by promoting the removal of damaged mitochondria [31]. Human and animal studies have shown that mitophagy regulators such as LC3II, PINK1, and Parkin have a reduced expression in all types of kidney cells (tubular cells, podocytes, mesangial cells, and endothelial cells) exposed to a high-glucose medium [2,124,125,126,127]. The study by Xiao et al. [128] revealed that the reduced expression of PINK1 and Parkin in the tubular cells of db/db mice was correlated with increased mROS production, mitochondrial fragmentation, and apoptosis. Similarly, a reduced expression of Parkin expression in tubular epithelial cells was seen in Streptozocin (STZ)-induced DKD [129].

There are also contrasting results: an increase in PINK1/Parkin pathway mitophagy in the kidneys of db/db mice [95] and stimulation of BNIP3-dependent mitophagy in kidney tubular cells after STZ-induced DKD [130]. These results may be due to a decreased clearance of mitophagosomes secondary to a defective mitophagy flux [130]. In peripheral blood mononuclear cells (PBMCs) from patients with DN, the mitochondrial metabolism is affected and is normal in diabetic patients without nephropathy. These data suggest that in DKD mitochondrial quality control is affected [131]. Diminished mitophagy activity was noted in renal biopsies of patients with DKD and in experimental mice models treated with high glucose [124]. The studies on diabetic mice revealed decreased expression of mitochondrial LC3II, Beclin1, PINK1, and Parkin and Atg5 in renal tissue which leads to reduced mitophagy activity [132]. The study by Chen et al. [133] observed the presence of inflammation, fibrosis, apoptosis, premature aging of renal tubular epithelial cells (RTECs), and decreased renal function in DN models with Parkin Knockout. Another study on diabetic mice reported increased ROS synthesis and, secondary, tubulointerstitial lesions induced by overexpression of a tubule-specific enzyme—MIOX which inhibits the PINK1/Parkin pathway of mitophagy [134].

All these data show that affected mitophagy can play a major role in the pathogenesis of DKD.

Mitochondrial dysfunction in mouse RTECs is not ameliorated by overexpression of PINK1 [9] but, in the case of overexpression of optineurin (OPTN)—a selective autophagy-adaptor protein, there is a decreased synthesis of mtROs, increased mitochondrial formation and activation of mitophagy which has like result amelioration of premature senescence of RTECs [124]. On the opposite side, reduced expression of OPTN seems to be associated with the accumulation of affected mitochondria and activation of the NLRP3 inflammasome which leads to high release of IL-18 and IL-1β and induces tubulointerstitial inflammation [135]. All these data raise the hypothesis that OPTN might be one of the most important protein regulators of mitophagy in RTECs [9].

In podocytes and in RTECs of diabetic mice there is a downregulation of PINK1, Parkin, LC3II/LC3I ratio, Mfn1 proteins, and an upregulation of p62 protein levels [9,127,134,136]. In a study on diabetic mice was described dilatation of podocytes and glomerular basement membrane thickening suggested that there is an inhibition of podocyte mitophagy influencing the progression of DN [137,138].

In patients with DKD and also in diabetic mice the expression of a secreted glycoprotein—PGRN was decreased in kidneys. The study by Zhou et al. [139] reported that downregulation of the PGRN can inhibit mitochondrial phagocytosis. In the kidney under high glucose conditions downregulation of the PGRN is associated with inhibition of the transcription factor FoxO1 which inhibits the expression of PINK1 and Parkin proteins and leads to mitochondrial disfunction [139].

Parkin-induced mitophagy can be promoted by the knockdown of lncRNA SNHG17 via regulating the degradation of Mst1 [125]. The conclusion of these studies is that different factors: PGRN, forkhead box class O1—FoxO1, and lncRNA SNHG17 influence PINK1/Parkin-mediated mitophagy by different pathways and promote DKD [9].

Regarding the mesangial cells, the study by Chen et al. [140] reported that in rat mesangial cells there is a high expression of LC3II/LC3I and p62 proteins—markers of autophagy and Parkin protein increased in affected mitochondria [141].

In STZ-induced type 1 DN the autophagy of mesangial cells implies activation of the PI3K/AKT/mTOR pathway [142]. Under high glucose conditions, there was a decreased mRNA expression of PINK1, Parkin, and Beclin1—proteins involved in an autophagic process (Zhang). Wen et al. [143] demonstrated that stimulation of mitophagy by mTOR/PINK1/Parkin pathway ameliorates renal inflammation, extracellular matrix deposition, and glomerular membrane thickening—Table 1. Other molecules such as ursolic acid, FBW7, and triptolide—TP influence autophagy and mitophagy and are involved in reno-protection [9].

The role of mitophagy in endothelial cells is evaluated in a study on db/db mice (an animal model of type 2 diabetes) which found that there is a decreased accumulation of LC3, ATP, and mtROS in glomeruli [127]. This study also demonstrated that there are decreased levels of PINK1, Parkin, and LC3II in mitochondrial glomerular endothelial cells exposed to high glucose. Activation of Nrf2 (nuclear erythroid 2-related factor 2)/ARE signaling and Torin1 activating factor of mitophagy plays important roles in mitophagy in glomerular endothelial cells [127].

In patients with type 2 diabetes (T2D), there is an impairment of mitophagy flux [6,126]. The studies of Czajka et al. [127] and Scheele et al. [144] observed that patients with prediabetes (with no important hyperglycemia) presented an increase in the expression levels of mitophagy genes: PINK1 and Parkin, NIX and patients with T2D had reduced expression of mitophagy-related genes. These data suggest that in prediabetes there is an increased mitophagy process in order to eliminate dysfunctional mitochondria, to avoid accumulation of altered mitochondria and aggravation of mitochondrial oxidative stress. In contrast, in T2D (characterized by higher levels of ROS) exists not only an increased mitochondrial damage but, also, there is a suppression of mitophagy which leads to accumulation of damaged mitochondria [145]. The results of all these studies suggest that mitophagy by the Pink1/Parkin pathway could be activated during early-stage diabetes in order to clear dysfunctional mitochondria from the kidney, but becomes overwhelmed as diabetic nephropathy progresses, leading to accumulation of damaged mitochondria and cell death [14]. However, the detailed regulatory mechanisms of mitophagy during the development of diabetic kidney disease remain largely unclear and further investigation is required to identify key regulators.

Bhatia and Choi [2] emphasize that autophagy and mitophagy are crucial for maintaining kidney homeostasis and regulating inflammatory responses in both acute and chronic kidney injuries [2].

### 1.7. Gaps in Knowledge

#### Heterogeneity in Autophagy Response

There is variability in how different kidney cell types respond to autophagy activation. Studies show that autophagy may have opposing effects depending on the cell type and disease stage. This cellular heterogeneity complicates efforts to develop targeted therapies that modulate autophagy in kidney diseases.

### 1.8. Regulation of Mitophagy in DN

Although it is known that mitophagy plays a role in DN, the specific regulatory mechanisms are still poorly understood. Some studies also showed that excessive mitophagy or long-term activation may lead to cell damage—this hypothesis needs to be searched in clinical trials [9]. More research is needed to clarify how mitophagy is triggered and controlled in response to hyperglycemia and mitochondrial dysfunction in DN and the role of mitophagy in the glomeruli and renal tubules in DKD. Jiang et al. [31] indicate that the PINK1/Parkin pathway is involved in mitophagy regulation in diabetic kidneys, but gaps remain in understanding how these pathways interact with other cellular stress responses [31]. Zuo et al. [14] highlight the need for further research on the cell-type-specific roles of mitophagy in kidney diseases [14].

In diabetic conditions, the mechanism underlying impaired mitophagy leading to glomerular lesions needs to be further investigated.

The recent advancements in investigative techniques of mitochondrial dysfunction in DKD are promising. The use of multiomics approaches, metabolic flux analysis, advanced imaging, mitochondrial morphometrics, and functional assessment provide very important information regarding mitochondrial activity, mitophagy, and metabolic alterations in DKD. For future directions of research, we take into account a notable innovation: the use of kidney organoids 3-dimensional multicellular structures derived from stem cells that mimic the structure and function of human kidneys. These organoids represent a more physiological model than cell cultures. High-resolution respirometry and advanced mass spectrometry techniques measure oxygen consumption rate and metabolite fluxes within these organoids [146]. Genetically encoded biosensors and advanced imaging technologies, like fluorescence lifetime imaging microscopy, allow real-time monitoring of metabolite levels and enzyme activities within living organoids [146]. These new methods facilitate the monitoring of precise manipulation and analysis of metabolic genes and can ensure deeper insights into mitochondrial function and roles in kidney physiology and pathology [146]. Multiomics technologies allow comprehensive analyses of mitochondrial function and can identify biomarkers of mitochondrial dysfunction. High-throughput genomics methods like whole-genome, single-cell, and single-nuclear RNA sequencing, uncover gene expression at the single-cell level to reveal cellular heterogeneity and the specific roles of kidney cells and immune cells in DN. Epigenetic and epi transcriptomic modifications introduce complexity to the elucidation of mitochondrial involvement in DN by regulating gene expression [146]. Integrating multiomics datasets with epigenetic and epi transcriptomic data can increase our understanding regarding their influence on mitochondrial function and metabolic reprogramming in DN. Proteomics and metabolomics offer important benefits in studying mitochondrial function, the tricarboxylic acid (TCA) or Krebs cycle, and oxidative phosphorylation (OXPHOS) in DKD. Proteomics facilitates (utilizing mass spectrometry) the identification and quantification of mitochondrial proteins and gives information about protein networks that regulate mitochondrial function [146]. Metabolomics enables a comprehensive analysis of metabolites and reveals metabolic alterations associated with DN. Advances in multiomics, imaging, metabolic research, and mitochondrial studies offer valuable information and can develop personalized therapeutic options (using verified biomarkers) [146].

### 1.9. Limitations in Current Research

#### 1.9.1. Limited Human Studies

Most studies on autophagy and mitophagy in DN and CKD are conducted in animal models or in vitro. While these studies provide valuable insights, there is a need for more clinical trials to validate findings in human patients.

Doblado et al. [6] point out that while animal models have advanced our understanding, the translation of mitophagy-targeting therapies to humans is still lacking [6].

There are important aspects of mitophagy modulation in humans that are not fully understood and research is necessary to identify new therapeutic targets. There are very few reliable and sensitive methods to study mitochondrial function, mitophagy, and autophagy in humans and these methods can be applied to limited tissues (that are accessible in humans): skin, skeletal muscle, and circulating blood cells. Also, these available methods are oriented on bioenergetics and do not evaluate other roles of mitochondria: amino-acid and vitamin metabolism, calcium homeostasis, steroid synthesis, and other roles that can be evolved in pathology. The recent availability of powerful and high-throughput technologies allows for measuring many proteins and metabolites in biofluids and could be utilized to develop autophagy–mitophagy profiles in clinical settings. New studies are needed to translate preclinical data on other circulating biomarkers such as extracellular vesicles. Nowadays, due to all these limitations, there are fewer observational studies and fewer interventional studies on mitophagy and mitophagy inducers in humans. Collaborations between clinicians and molecular biologists are mandatory to understand mitophagy and autophagy roles in human pathology and physiology and to elaborate therapeutic strategies [147].

#### 1.9.2. Context-Dependent Effects of Autophagy

Autophagy and mitophagy can have both protective and detrimental effects depending on the disease stage and cellular environment. This duality makes it challenging to design therapeutic interventions that consistently provide benefits across different stages of CKD and DN. Saxena et al. [32] discuss the complexities of autophagy regulation and its context-dependent effects, which must be addressed in future therapeutic approaches. The roles of autophagy during kidney fibrosis are possibly to be context-dependent and temporal and need to be further evaluated [2]. In patients with type 1 diabetes (T1DM) and type 2 diabetes (T2DM), there is an inhibition of autophagy in islet β-cells [148] so, by inducing autophagy it can improve the β-cells dysfunction in DM. In T1DM, functional islet β-cell mass is reduced to <10% of normal levels, impairing insulin secretion. In the early stages of T1DM, autophagy is paradoxically overactivated by high glucose-induced oxidative stress and has consequences such as lysosome depletion and autophagy deficiency [148]. This deficiency aggravates oxidative stress and mitochondrial damage, further leading to lysosomal lesions and β-cell apoptosis. In T2DM insulin resistance (IR) is a key mechanism and is influenced by autophagy, which regulates β-cells and insulin-responsive tissues, such as the adipose tissue, liver, and skeletal muscle [148,149]. Yang et al. demonstrate in a study with high-fat diet (HFD)-fed rats that inducing autophagy can alleviate IR by stabilizing insulin receptor substrate-1(IRS-1)—a key mediator of insulin signaling [148,150]. The overproduction of nitric oxide (induced by inflammation in the liver which leads to steatosis—a sign of liver IR) can impair lysosomal function and affect cell’s ability to remove damaged cellular components [148].

Sakai et al., 2019 [148] found in a study that the level of autophagy in proximal tubular epithelial cells varies depending on the stage of the disease: in the early stage of high glucose (HG) treatment the autophagy increases but, later, suppressed due to lysosomal stress (induce by excessive autophagy). The activation of the mTOR pathway in mice with T2DM induces the suppression of autophagy and this mechanism is not observed in mice with T1DM. The conclusion of these findings is that there are differences in the mechanisms of autophagy suppression between T1DM and T2DM. In T2DM autophagy suppression is due to decreased lysosomal activity while in T1DM is due to lysosomal depletion (induced by excessive initial activation of autophagy). Rapamycin (promoter of autophagy) is administered in mice with T1DM in the early stage of the disease increases their kidney damage but can diminish kidney lesions in mice with T2DM. These findings show that in the early stages of T1DM autophagy is overactivated to avoid kidney damage and further stimulation of autophagy increases lysosomal stress and accelerates disease progression [148].

### 1.10. Future Research Directions

#### 1.10.1. Targeted Therapies for Autophagy and Mitophagy Modulation

Future studies should focus on identifying specific molecular targets within the autophagic and mitophagy pathways that can be modulated to treat DN and CKD. Recent studies suggest that targeting nutrient-sensing pathways like AMPK, mTOR, and Sirt1 could hold promise for new treatments—Table 1. Rapamycin, resveratrol, and quercetin are mitophagy inducers and can be protective molecules against DKD [9]—Figure 3. Rapamycin—an inhibitor of mTOR stimulates autophagy and mitophagy by restoring the expression of PINK1 and Parkin [9]. Rapamycin ameliorates the short-term pathological lesions in diabetic nephropathy in animal models (glomerular hypertrophy, renal inflammation, interstitial fibrosis) by blocking the mTORC1/ULK1 pathway and increasing autophagy [37]. Overactivation of mTOR can cause insulin resistance in humans and has been implicated in type 2 diabetes [151]. The studies demonstrate that Rapamycin prevents insulin resistance induced by nutrient infusion in humans, reduces insulin resistance in diabetic and hyperinsulinemic rats, and normalizes glucose metabolism in diabetic mice [151]. Rapamycin can prevent or ameliorate diabetic nephropathy in both rats and mice. Hyperactivation of mTOR in podocytes leads to diabetic nephropathy and premature death in mice, which can be preventable by treatment with rapamycin [151]. In some studies, chronic administration of rapamycin-induced insulin resistance or glucose intolerance without insulin resistance, but also insulin sensitization with glucose intolerance. In many other studies, chronic daily administration and, especially, intermittent or low-dose administration of rapamycin did not cause hyperglycemia. The effects of rapamycin depend on its dose, duration of administration (especially), time and route of administration, species and/or strain, sex, diet, obesity, and other factors. For the outcome, the beta cell function is very important. The insulin resistance observed in mice during treatment can be explained by prolonged deactivation of mTORC2 [151]. Insulin resistance (due to mTORC2 deactivation) in addition to decreased insulin secretion (due to mTORC1 deactivation) may contribute to glucose intolerance [151]. As we commented previously, rapamycin (autophagic inducer) alleviated diabetic nephropathy in type 2 DM but aggravated it in type 1 DM [148]. There are studies that discovered that excessive autophagy has harmful effects: aggravates muscle atrophy induced by DN, and induces autophagic cell death and myocardial damage in diabetic cardiomyopathy [148]. To establish the optimal dosage for autophagy induction therapy and rigorously evaluate its side effects are needed future research.

To date, Rapamycin use is limited in long-term clinical treatment due to important side effects (anemia, decreased blood platelets, infections, arterial hypertension, and so on).

Studies showed that if resveratrol activates the expression of BNIP3, Sirt, and Beclin 1 [146], quercetin reduces the aging of renal tubular epithelial cells via the SIRT1/PINK1 mitophagy pathway [122] and coenzyme Q10 (CoQ10) activates mitophagy by restoring antioxidant response element—Nrf2 in diabetic nephropathy [127,152].

In a study with a mouse model of type 1 diabetes treatment with Mitoquinone (MitoQ) increased mitophagy regulators ameliorated tubular and glomerular functions and diminished interstitial fibrosis [153,154,155].

Metformin (an AMPK agonist) activates mitophagy and reduces oxidative stress and fibrosis in STZ-treated diabetic mice and in high glucose/high fatty acid-treated tubular epithelial cells [136]. Metformin preserved mitochondrial health in the mononuclear cells of patients with type 2 diabetes by increasing the expression of the gene of mitophagy: PINK1, Parkin, LC3, and NIX [6]. Due to its capacity to activate mitophagy, metformin may be able to stretch far beyond obesity and type 2 diabetes.

Administration of therapeutic mitophagy inducer alone in patients should be prudent [14] because there is a balance between mitophagy which decreases mitochondrial quantity by removing damaged mitochondria and mitochondrial biogenesis which elevates mitochondrial mass in order to replace the mitochondria that have been removed by mitophagy and/or to accomplish higher energy demand. In our days is not known if mitophagy can be specifically targeted [14]. However, the search for inducers of mitophagy with minimal side effects is mandatory.

Lu et al. [95] reported in a study that Ursolic acid attenuates diabetic mesangial cell injury through the up-regulation of autophagy via miRNA-21/PTEN/Akt/mTOR suppression.

In a study with subtotal nephrectomized rats, activation of autophagy by Tubastatin A was associated with reductions in tubulointerstitial fibrosis and tubular apoptosis [151].

Inhibitor of SGLT2—Empagliflozin attenuates diabetic tubulopathy by improving mitochondrial fragmentation and autophagy [156]. SGLT2 inhibitor Dapagliflozin suppresses renal inflammation, apoptosis, and ER stress and by these actions delay the progression of renal complications in pre-diabetes. SGLT2 inhibitors have been used in clinical trials and demonstrate their protective effect in DN [36]—Table 1.

Other hypoglycemic agents such as glucagon-like peptide 1 receptor (GLP-1R) and dipeptidyl peptidase-4 (DPP-4) inhibitors have been shown in experimental studies that could modulate autophagy [37,155]—Table 1. Experimental studies with human proximal tubular epithelial cells (PTECs—HKC-8) exposed to AGEs and with Zucker diabetic fatty rats demonstrated that liraglutide (a GLP-1R agonist) activates autophagy and reduces oxidative stress via AMPK/mTOR pathway and improve the prognosis of DKD [156,157,158]. Another study on db/db mice reported that SGLT2 inhibitor empagliflozin and DPP-4 inhibitor linagliptin reactivate glomerular autophagy and ameliorates glomerular morphology: alleviate podocyte foot process effacement and mesangial expansion and reduce albuminuria [159,160]—Table 1.

A novel therapeutic target for CKD treatment can become the exosome—a type of extracellular vesicles that carry different biomolecules and is involved in intercellular communication [161]. MiRNAs contained in the exosome promote autophagy and diminish high glucose-induced renal cell injury [162].

Activation of PGRN (progranulin) and OPTN (optineurin) could be another novel therapy for preventing and treatment of DN [14,135,139].

Han et al. [36] recommend exploring autophagy modulators that could restore balance in autophagic activity in DN.

Future research should focus on determining the optimal dosage for autophagy induction therapy and mitophagy inducer and evaluating side effects. Excessive dosages of drugs may cause cell damage and worsen the disease. It must be taken into account that there is a species-specific nature of many drugs (those effective in mice but not in humans and vice versa) and complicates direct translation to human therapy. Also, factors like sex, age, and the presence of other diseases may influence drug efficacy. A large number of randomized, double-blind, and placebo-controlled clinical trials are mandatory to investigate optimal dosage, efficacy, long-term effects, and side effects of drugs [148]. Future research also needs to investigate how autophagy and mitophagy interact with cellular environmental disorders: endoplasmic reticulum stress and oxidative stress.

#### 1.10.2. Human Clinical Trials

More human studies are needed to validate the therapeutic potential of autophagy and mitophagy modulators in DN and CKD. Researchers should prioritize translational research that bridges the gap between preclinical findings and clinical applications. Zuo et al. call for more clinical trials that assess the safety and efficacy of mitophagy-targeting drugs in human populations [14].

## 2. Conclusions

Autophagy and mitophagy are critical processes in the progression and treatment of diabetic nephropathy and chronic kidney disease. While substantial progress has been made in understanding these mechanisms, many gaps remain, particularly regarding the heterogeneity of cellular responses, the regulation of mitophagy, and the translation of findings from animal models to human clinical trials. Future research should aim to develop targeted therapies that can modulate these pathways to mitigate the effects of DN and CKD. This review tries to synthesize recent knowledge in the field and to point out the main pathways that need further research, to translate from preclinical to clinical trials and discover new therapeutic agents.

## Figures and Tables

**Table 1:** Major roles of Mitophagy and Autophagy in diabetic nephropathy.

Autophagy in DN		
Key Molecules/Targets	Roles	Mechanisms
AMPK	Alleviates damage associated with DN	Induces autophagy by inhibiting the mTOR pathway. In DN mouse models the expression of AMPK is significantly suppressed. Increasing the expression of AMPK can ameliorate the lesions in the DN.
mTOR (mTOR1 and mTOR2)	Inhibition of mTOR alleviates the lesions in DN.	Overexpression of mTOR is associated with IR and the inhibition of autophagy so, inhibition of mTOR can ameliorate the lesions in DN.
SIRT1	Activation of SIRT1 can ameliorate renal autophagy activity, increase mitochondrial regeneration, reduce oxidative stress and inflammation, and improve blood glucose homeostasis and IR.	SIRT1 stimulates autophagy by different pathways: modulation of autophagy activity, antioxidant stress, and anti-aging mechanisms.
SGLT-2	SGLT-2 inhibitors protect against DN	SGLT-2 inhibitors (such as dapagliflozin) can activate autophagy, reduce oxidative stress and apoptosis induced by hyperglycemia
GLP-1	Ameliorate IR and lesions in DN	GLP-1 receptor agonists (such as liraglutide) activate autophagy
DPP-4	Ameliorate kidney function and blood glucose control	DPP-4 inhibitors (such as sitagliptin) can stimulate autophagy
**MITOPHAGY IN DN**		
**Key Molecules/Targets**	**Roles**	**Mechanisms**
PINK1/Parkin	Protects against DN, prevents fibrosis	Increase mitophagy, reduce oxidative stress, promote clearance of damaged mitochondria, and ameliorate kidney function.
AMPK	Increases mitophagy, protects against fibrosis and DN	Regulates BNIP3 phosphorylation, phosphorylates ULK1
mTOR	Protects against DN	Inhibition increases mitophagy, promotes the interaction between AMPK and ULK1, and reduces renal tubular cell lesions.
SIRT1	Regulates mitophagy, acts against kidney fibrosis	Stimulates mitophagy through the PINK1/Parkin pathway reduces oxidative stress, and ameliorates mitochondrial function
Nrf2	Induces mitophagy and protects against fibrosis and DN	Increase clearance of damaged mitochondria, reduce oxidative stress
NIX (BNIP3L)	Protects against mitochondrial dysfunction and fibrosis	Stimulates mitochondrial depolarization and ROS generation and induces mitophagy

Abbreviations: AMPK adenosine 5′-monophosphate-activated protein kinase, DN diabetic nephropathy, DPP-4 dipeptidyl-peptidase 4, GLP-1 glucagon-like-peptide-1, IR insulin resistance, mTOR mammalian target of rapamycin, SGLT-2 sodium-glucose cotransporter 2, SIRT1 sirtuin 1, BNIP3L Bcl-2 homology 3 (BH3)-only protein Nix, Nrf2 nuclear factor erythroid 2-related factor 2.
